# Causal association of metformin and osteoporosis: A 2-sample Mendelian randomization study

**DOI:** 10.1097/MD.0000000000035191

**Published:** 2023-10-27

**Authors:** Yong-Kang Wei, Ping-Bo Chen, Ling-Ling Ju, Guang-Hua Deng

**Affiliations:** a The Fourth Clinical Medical College of Xinjiang Medical University, Urumqi, Xinjiang, China; b Xinjiang Uygur Autonomous Region Hospital of Traditional Chinese Medicine, Urumqi, Xinjiang, China; c Institute of Biomedical Engineering, College of Medicine, Southwest Jiaotong University, Chengdu, Sichuan, China; d Ya’an City Hospital of Traditional Chinese Medicine, Ya’an, Sichuan, China.

**Keywords:** Mendelian randomization, metformin, osteoporosis

## Abstract

To investigate the causal relationship between metformin use and osteoporosis and different subtypes of osteoporosis using a 2-sample Mendelian randomization method. Data from genome-wide association studies were analyzed, with the exposure factor being metformin and the outcome variables being osteoporosis and different subtypes. Mendelian randomization was performed using Inverse Variance Weighted (IVW), MR-Egger, and weight median (WM) methods, and heterogeneity tests, horizontal multivariate analyses, and sensitivity analyses were performed. The IVW method analysis with metformin and osteoporosis showed *P* = 1.53E-04, OR (95%CI) = 1.81E-02 (2.27E-02-1.44E-01); the IVW method analysis with metformin and postmenopausal osteoporosis with pathologic fracture showed *P* = 2.22E-01, OR (95%CI) = 4.89E-02 (3. 83E-04-6.23E + 00); the IVW method using metformin with osteoporosis with pathological fracture showed that *P* = 2.14E-01, OR (95%CI) = 1.64E + 00(5.78E-02-6.44E-04); the IVW method using metformin with pharmacological osteoporosis with pathological fracture showed that *P* = 9. 83E- 01, OR (95%CI) = 1.11E + 00 (3.99E-05-3.11E + 04); IVW method of metformin use and pharmacological osteoporosis showed that *P* = 5.99E-01, OR (95%CI) = 2.27E + 01 (2.00E-04-2.57E + 06); there is a causal relationship between metformin use and osteoporosis, but there is no causal relationship between metformin use and postmenopausal osteoporosis with pathological fracture, osteoporosis with pathological fracture, pharmacological osteoporosis, and pharmacological osteoporosis with pathological fracture, and metformin use is a protective factor for osteoporosis.

## 1. Introduction

Osteoporosis, one of the most common degenerative diseases in orthopedics, is particularly prevalent in the middle-aged and elderly population, especially in middle-aged and elderly women, and its incidence increases with age. 50% of women will develop osteoporosis after age 50, and approximately 30% of men will develop osteoporosis after age 50.^[1,2]^ For women over 55 and men over 65, osteoporotic fractures and the pain associated with disease progression significantly impact the quality of life of middle-aged and elderly patients.^[3]^

The development of osteoporosis is associated with various factors, including endocrine, genetic, pharmacological, and disuse factors, as well as various risk factors such as age, gender, obesity, rheumatoid arthritis, and diabetes mellitus. However, with the development of medicine and the clinical application of various interventions, the current situation of the development of osteoporosis has been improved.^[4,5]^ However, osteoporosis remains a common orthopedic chronic disease threatening health.

Diabetes mellitus is the most common endocrine disease, and it has been clinically observed that patients with diabetes mellitus have a higher probability of developing osteoporosis, and studies have been reported to confirm that diabetes mellitus is a risk factor for osteoporosis.^[6]^ Type 2 diabetes mellitus can directly affect bone metabolism and bone strength,^[7]^ in addition to lower vitamin D levels in patients with type 2 diabetes mellitus,^[8]^ and these changes lead to an increased incidence of osteoporosis in patients with diabetes mellitus. Therefore, prevention and treatment of osteoporosis in diabetic patients at high risk for osteoporosis appear essential. Metformin, as an insulin sensitizer, is a first-line drug for the treatment of diabetes and has recently been shown to have other roles in addition to its role in the treatment of diabetes, among which its role in osteoporosis deserves our attention.^[9]^

Although evidence from randomized controlled trials is still lacking, relevant basic studies have demonstrated a protective effect of metformin on the development of osteoporosis in mice, and some clinical studies have provided some evidence of an association between metformin and osteoporosis.^[10,11]^ Mendelian randomization studies, a reliable epidemiological research method that uses genes as instrumental variables to prove causality by eliminating confounding interferences, are stronger than observational studies and second to randomized controlled trials in evidence-based medicine, which happens to provide further evidence for the association between metformin and osteoporosis.^[12]^ Therefore, the present study was conducted to further investigate the causal relationship between metformin use and osteoporosis using the 2-sample Mendelian randomization method based on publicly available relevant data from UK Biobank and FinnGen to provide more reliable evidence on the possible role of metformin on osteoporosis.

## 2. Material and methods

### 2.1. Study design

This study used a 2-sample Mendelian randomization method to explore the causal association between metformin use and the risk of developing osteoporosis. Exposure factors were: treatment/medication code: metformin. Outcome variables were: osteoporosis, Postmenopausal osteoporosis with pathological fracture, Osteoporosis with pathological fracture, Drug-induced osteoporosis with pathological fracture, and Drug-induced osteoporosis with pathological fracture.

### 2.2. Data source

Information on genetic variants involved in this study was obtained from publicly available GWAS (genome-wide association studies), and phenotypic definitions were consistent with this study. Exposure factors were obtained from the UK Biobank (UK Biobank, version 3, March 2018), and outcome variables were obtained from the FinnGen (FinnGen round 9).

FinnGen, a large GWAS study based on the complete health and genetic information of 500,000 Finnish biobank participants, has been updated with 11 rounds as of August 1, 2023, with the most recent publicly available data being round 9. The 2 samples were independent without overlap, and the populations were European.

Since its inception in 2006, the UK Biobank has collected blood, urine, and saliva samples from 500,000 participants across the UK and established demographic, socioeconomic, lifestyle, and health information, making it one of the most comprehensive aggregated databases of GWAS studies. The data used in this study comes from another GWAS study published by UKB in 2018.

A total of 11,552 Treatment/medication code: metformin cases and 451,381 controls were sequenced in a European population with T Treatment/medication code: metformin as an exposure factor, including 9851,867 single-nucleotide polymorphisms (SNPs). The result was a European population diagnosed with osteoporosis with 7300 positive cases and 358,014 controls, sequenced with 20,169,594 SNPs. The European population with postmenopausal osteoporosis and pathologic fracture, with 1351 positive cases and 209,313 controls, was sequenced with 20,162,410 SNPs. The European population diagnosed with drug-induced osteoporosis with pathologic fracture had 357 positive cases and 375,618 control cases, with 20,170,214 SNPs sequenced. The European population with drug-induced osteoporosis had 289 positive cases and 376,988 controls, with 20,170,232 SNPs sequenced. All GWAS data and tables related to the instrumental variable rsID are available upon request from the authors. Details are provided below in Table 1.

**Table 1:** Sample information.

Exposure/outcome	Yr	Population	Sample size	Number of SNPs	Data from
Treatment/medication code: metformin	2018	European	11,552/ 451,381	9851,867	UKB
Osteoporosis	2023	European	7300/358,014	20,169,594	FinnGen
Postmenopausal osteoporosis with pathological fracture	2023	European	1351/209,313	20,162,410	FinnGen
Osteoporosis with pathological fracture (FG)	2023	European	1659/285,030	20,167,428	FinnGen
Drug-induced osteoporosis with pathological fracture	2023	European	357/375,618	20,170,214	FinnGen
Drug-induced osteoporosis	2023	European	289/376,988	20,170,232	FinnGen

SNP = single nucleotide polymorphism.

All data for this study were obtained from the publicly available GWAS study database and therefore did not require ethical approval.

### 2.3. Instrumental variables filter

A genome-wide significance threshold of *P* < 5E-8 was set based on previous reliable research studies, the linkage disequilibrium parameter (r2) was set to 1E-3, and the genetic distance for clumping was set to 10MB to screen out instrumental variables with no linkage effect as SNPs with correlation with metformin use, and PhenoScanner (http://www.phenoscanner.medschl.cam.ac.uk/) was queried to remove SNPs with correlation with outcome from the screened SNPs, respectively.^[13]^ Using the formula F = beta^2^/se^2^, the statistical power F.^[14]^ SNPs screened by the tool were considered strong instrumental variables when F-statistic > 10. The specifics of the instrumental variables can be found in the Supplementary Material. http://links.lww.com/MD/K389

### 2.4. Data analysis

All statistical analyses and plots in this study were done in Rstudio software based on R version 4.3.0, and data analyses were mainly done based on the “TwoSampleMR” R package.

The 2-sample Mendelian randomization analysis was performed with Inverse variance weighted as the main method, supplemented by MR Egger and Weighted Median. Cochrane Q test, leave-one-out test and MR-Egger intercept test were used for heterogeneity analysis. Intercept test, heterogeneity test, sensitivity analysis, and horizontal multiple validity test. To avoid excessive β-error due to multiple comparisons, a correction for multiple comparisons, the Bonferroni correction, was used, that is, *P* < .05/E/B (E is the number of exposure factors and B is the number of outcome variables).^[15]^

MR analysis is based on the genetic information of SNPs without pleiotropy, and weighted inverse variance is the main analytical method of MR, which provides the most accurate results of causal linkage when pleiotropy is nonexistent.^[16]^

As complementary methods, MR Egger and Weighted Median are equally important, and concerning previous studies, these 2 methods are of great significance for MR analysis. These 2 methods are mainly used to analyze the results when heterogeneity and pleiotropy are present. In the nonexistence of pleiotropy and heterogeneity, we preferred to use IVW results for interpretation. However, we can also interpret WM results when there is heterogeneity without pleiotropy. And when there is pleiotropy, it is necessary to use the results calculated by the MR-Egger method.^[17]^ If the results of the IVW method are significant, the effect values beta of the 2 complementary methods are consistent with the IVW method. If there is no pleiotropy, the results can be judged as positive regardless of the significance of the *P* values of the other 2 complementary methods.^[18]^

## 3. Results

After the screening, excluding 42 SNPs as instrumental variables in postmenopausal osteoporosis with pathological fracture, with all other exposures, there were 43 SNPs as instrumental variables satisfying the 3 main hypotheses. Statistical strength F-values were calculated, and all F-values were >10, indicating that the instrumental variables examined were strong instrumental variables. Significance threshold after Bonferroni correction *P* < .01.

In the MR analysis of metformin treatment and osteoporosis, the IVW method, MR-Egger method, WM method, simple mode method, and weighted mode method analyses were in the same direction, with *P* = 1.53E-04 for IVW, a value <0.01, indicating a strong correlation between metformin treatment and osteoporosis. And in the heterogeneity test, the *P* values of Cochrane Q test (IVW and MR-Egger methods) were >.05, so there was no heterogeneity. In the leave-one-out test, no SNPs were found to have a strong effect on the causality estimates, and the P = O.819 for the horizontal multiplicity test (MR-Egger intercept test) was >0.05, so it can be concluded that there is no horizontal multiplicity.

**Figure 1.**
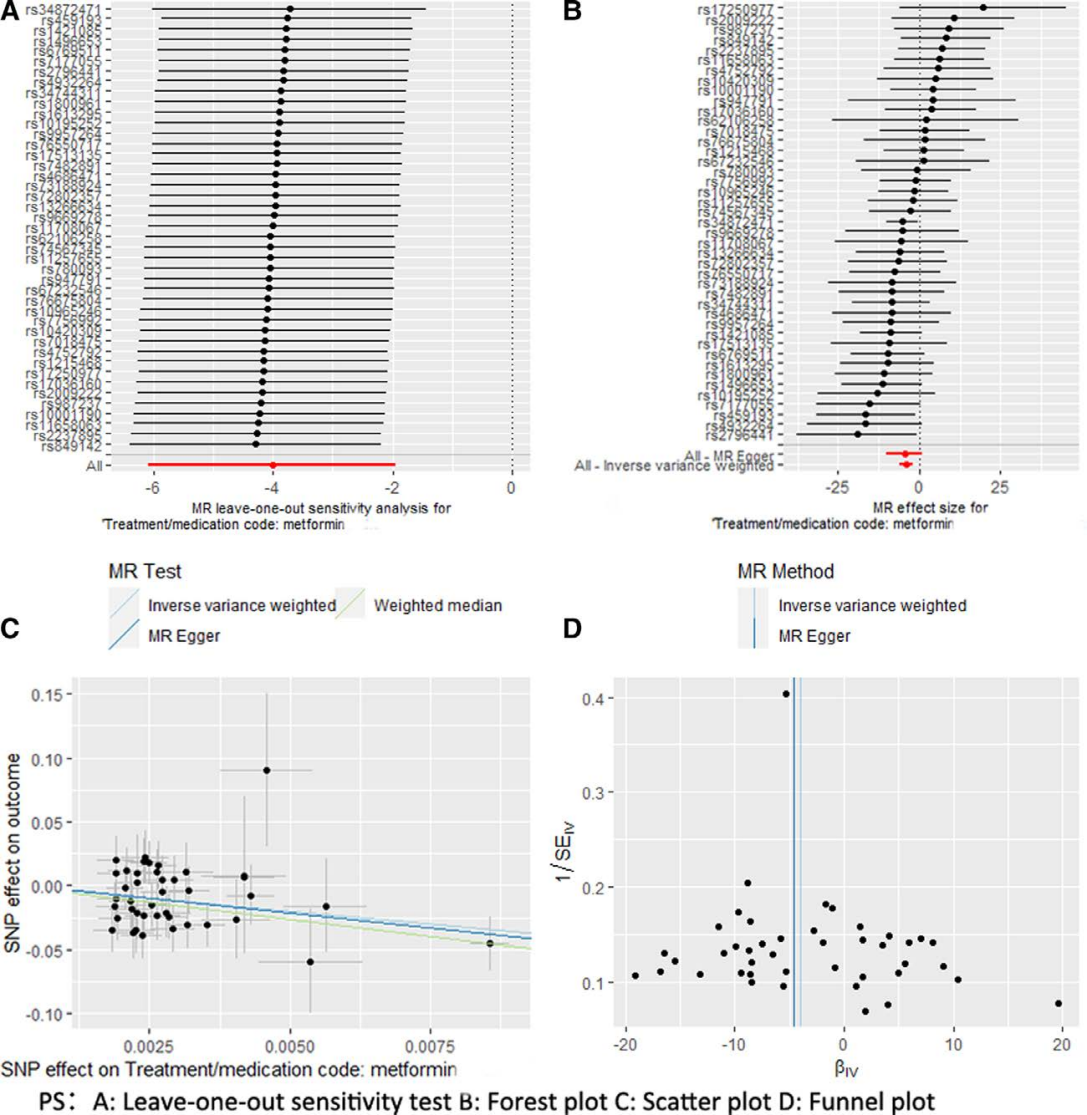
Visualization of Mendelian randomization analysis using metformin and osteoporosis.

There was no heterogeneity and horizontal pleiotropy in the MR analysis of metformin use with all other osteoporosis subtypes. The IVW method, MR-Egger method, WM method, Simple Mode method, and Weighted Mode method analyses were in the same direction. Still, the *P* values for the IVW method were all >.01 or 0.05, so the use of metformin with postmenopausal osteoporosis with pathological fracture, osteoporosis with pathological fracture, pharmacological osteoporosis with pathological fracture, and pharmacological osteoporosis did not have a significant causal relationship. Detailed results and visualization are provided below in Tables 2, 3, and Figure 1.

**Table 2:** Mendelian randomization analysis of the main results.

Outcome	SNPs	Method	OR (95%CI)	*P*
Osteoporosis	43	IVW	1.81E-02 (2.27E-02-1.44E-01)	1.53E-04
		MR Egger	9.78E-03 (3.54E-05-2.71E + 00)	1.14E-01
		Weighted Median	5.03E-03 (1.80E-04-1.41E-01)	1.85E-03
Postmenopausal osteoporosis with pathological fracture	42	IVW	4.89E-02 (3.83E-04-6.23 + 00)	2.22E-01
		MR Egger	1.25E-04 (2.40E-10-6.53E + 01)	1.88E-01
		Weighted Median	4.65E-03 (3.26E-06-6.64E + 00)	1.38E-01
Osteoporosis with pathological fracture (FG)	43	IVW	1.64E + 00(5.78E-02-6.44E-04)	2.14E-01
		MR Egger	1.26E-02 (5.82E-08-2.73E + 03)	4.89E-01
		Weighted Median	2.43E-03 (2.92E-06-1.87E + 00)	7.57E-02
Drug-induced osteoporosis with pathological fracture	43	IVW	1.11E + 00(3.99E-05-3.11E + 04)	9.83E-01
		MR Egger	7.04E + 06(8.32E-06-5.97E + 18)	2.67E-01
		Weighted Median	1.81E + 00(6.02E-07-5.42E + 06)	9.38E-01
Drug-induced osteoporosis	43	IVW	2.27E + 01(2.00E-04-2.57E + 06)	5.99E-01
		MR Egger	1.66E + 03 (2.51E-11-1.09E + 17)	6.50E-01
		Weighted Median	2.92E + 06(4.81E-01-1.77E + 13)	6.18E-02

Exposure is Treatment/medication code: metformin.

IVW = inverse variance weighted, MR = Mendelian randomization, OR = odds ratio, SNP = single nucleotide polymorphism.

**Table 3:** Testing for heterogeneity and horizontal pleiotropy.

Outcome	Method	Q_pval	Method	pval
Osteoporosis	MR Egger	4.69E-01	pleiotropy-test	8.19E-01
	IVW	5.11E-01		
Postmenopausal osteoporosis with pathological fracture	MR Egger	4.09 E-01	pleiotropy-test	3.45 E-01
	IVW	4.11 E-01		
Osteoporosis with pathological fracture (FG)	MR Egger	2.93 E-01	pleiotropy-test	7.95 E-01
	IVW	3.29 E-01		
Drug-induced osteoporosis with pathological fracture	MR Egger	1.41 E-01	pleiotropy-test	2.36 E-01
	IVW	1.27 E-01		
Drug-induced osteoporosis	MR Egger	7.32 E-02	pleiotropy-test	7.78 E-01
	IVW	8.74 E-02		

IVW = inverse variance weighted, MR = Mendelian randomization.

## 4. Discussion

The association between metformin and osteoporosis has been proposed for a long time, but at present, it is only in the preclinical stage, and there are still many confounding factors, making the association of metformin with osteoporosis doubtful. In this study, the causal relationship between metformin use and osteoporosis and various subtypes of osteoporosis was investigated using a 2-sample Mendelian randomization method, excluding confounding factors and using genes as instrumental variables.

Based on the study results, we can conclude that the use of metformin is a protective factor for osteoporosis and that the use of metformin has a causal relationship with osteoporosis. However, metformin use was not causally associated with all types of osteoporosis. Metformin is not causally associated with all types of osteoporosis in which pathologic fractures occur, nor is it associated with all types of pharmacologic or postmenopausal osteoporosis.

Meta-analysis suggests a protective effect of metformin against osteoporosis.^[19]^ A retrospective, single-center study of 11,458 Chinese patients confirmed that metformin use was associated with increased bone mineral density.^[20]^ In a large island-wide retrospective cohort study of 7827 participants in Taiwan, we learned that the incidence of osteoporosis was lower in diabetic patients using metformin, OR (95%) = 0.820 (0.691–0.972).^[21]^ This confirms the results of our study.

The mechanism of how metformin works in the body to prevent or alleviate osteoporosis in diabetic patients is poorly understood. Still, it is generally believed to act on osteoblasts and osteoclasts to achieve a protective effect on bone mass. In 2008, Japanese researchers investigated the mechanism of action of metformin and osteoporosis using a cloned osteoblast cell line isolated from mouse cells. Metformin activates the AMPK pathway to promote osteoblast differentiation.^[22]^ In recent years, with the progress of biological science, more and more basic studies based on mice have explained the mechanism of metformin effect on osteoporosis at the molecular level: metformin inhibits E2F1-mediated autophagy of osteoclast precursors to reduce bone loss, metformin reverses oxidative stress-induced apoptosis of osteoblast precursor cells through the EGFR/GSK-3β/calcium pathway to preserve more osteoblasts and protects the differentiation potential of osteoblasts in high glucose environment. It also protects the differentiation potential of osteoblastic stem cells in high glucose environment and activates the Nrf2/HO-1 signaling pathway to regulate the inhibition of osteogenic differentiation caused by high glucose; in addition, metformin can reverse the apoptosis caused by glucocorticoids through the action of AMPK/mTOR/p70S6K pathway on osteoblastic cells, thus playing a role in pharmacological osteoporosis.^[23–27]^ More specific mechanisms are being investigated in further studies.

Metformin, as a first-line glucose control agent, has certain limitations when used as an osteoporosis prevention and treatment agent. Metformin, as an anti-osteoporosis drug in osteoporosis patients without diabetes mellitus, may lead to hypoglycemia, which may be life-threatening. In addition, the use of metformin in diabetic patients also carries certain medication risks, as metformin may lead to DRESS syndrome,^[28]^ an imbalance of the intestinal microflora, and other adverse effects in addition to those documented in the specification.^[29]^ It is worth noting that among diabetic patients using metformin for glycemic control, female patients are more prone to adverse effects.^[30]^ As for patients with osteoporosis, women predominate, so the benefit of using metformin for female osteoporosis patients with comorbid diabetes needs to be considered more carefully.

In conclusion, although metformin has a certain protective effect on osteoporosis, a series of questions, such as whether it can be applied in the clinic, whether it can be used for a long time, and what kind of population it is suitable for, need to be answered by more evidence from further studies.

The present 2-sample Mendelian randomization study elucidated the causal relationship between metformin use and osteoporosis. Mendelian randomization was used to minimize confounding and avoid reverse causality. Data were obtained from 2 of the most credible genetic databases and European populations, with independent samples to avoid bias due to ethnicity and confounding due to overlapping exposure and outcome samples. The large sample size of the study enhances its value.

The ICD-10 diagnostic classification does not provide an exhaustive classification of all types of osteoporosis, and therefore the classification subtypes are not complete; the small positive sample size for drug-related osteoporosis and drug-related osteoporosis with a pathological fracture in the study outcome reduces the statistical adequacy of the results to some extent; the study population is European, and therefore the conclusions of this study may have some limitations. The applicability of the results of this study may be limited. In addition, the results of the Mendelian randomization study could only establish the causal relationship between exposure and outcome, and further investigation of the biological mechanisms of metformin use and osteoporosis could not be conducted.

## 5. Conclusion

In conclusion, we confirmed the causal relationship between metformin use and osteoporosis using gene-level association with the Mendelian randomization method. We proved that metformin use is a protective factor for osteoporosis. Still, there was no significant association between metformin use and the outcome of each type of osteoporosis with pathological fracture. This provides stronger evidence for the use of metformin in the prevention and treatment of osteoporosis in diabetic patients, and together with more basic experiments and clinical trials, the protective effect of metformin on osteoporosis will surely be emphasized in the future, and the glycemic control program for diabetic patients will surely be improved.

## Author contributions

**Conceptualization:** Yong-Kang Wei.

**Formal analysis:** Guang-Hua Deng.

**Methodology:** Ping-Bo Chen, Ling-Ling Ju.

**Writing – original draft:** Yong-Kang Wei.

**Writing – review & editing:** Yong-Kang Wei.

## Supplementary Material


