# Supplementary material for: Causal association of metformin and osteoporosis: A 2-sample Mendelian randomization study
Source: Medicine (Baltimore). 2023 Oct 27;102(43):e35191. doi: 10.1097/MD.0000000000035191 (PMC10615397; doi:10.1097/MD.0000000000035191)
Supplement: Supplementary file 1 [file medi-102-e35191-s001.docx]

| SNP | beta | samplesize | pval | se | chr | pos | id | effect_allele | other_allele | eaf | R | F |
| --- | --- | --- | --- | --- | --- | --- | --- | --- | --- | --- | --- | --- |
| rs17513135 | 0.002275 | 462933 | 3.70E-09 | 0.000386 | 1 | 40035686 | ukb-b-14609 | T | C | 0.227468 | 0.008666 | 34.76479 |
| rs1515096 | 0.002544 | 462933 | 9.40E-13 | 0.000356 | 2 | 2.27E+08 | ukb-b-14609 | T | C | 0.426595 | 0.010492 | 50.9639 |
| rs62106258 | -0.00419 | 462933 | 2.60E-08 | 0.000752 | 2 | 417167 | ukb-b-14609 | C | T | 0.04854 | -0.00818 | 30.95322 |
| rs10195252 | -0.00192 | 462933 | 6.10E-09 | 0.00033 | 2 | 1.66E+08 | ukb-b-14609 | C | T | 0.405055 | -0.00855 | 33.81789 |
| rs780093 | 0.002063 | 462933 | 5.10E-10 | 0.000332 | 2 | 27742603 | ukb-b-14609 | C | T | 0.615229 | 0.009134 | 38.62204 |
| rs76675804 | -0.00418 | 462933 | 8.80E-15 | 0.00054 | 2 | 43611883 | ukb-b-14609 | C | T | 0.100098 | -0.0114 | 60.15124 |
| rs4686471 | 0.001889 | 462933 | 1.40E-08 | 0.000333 | 3 | 1.88E+08 | ukb-b-14609 | C | T | 0.610082 | 0.008335 | 32.16555 |
| rs11708067 | -0.00217 | 462933 | 9.50E-09 | 0.000377 | 3 | 1.23E+08 | ukb-b-14609 | G | A | 0.242368 | -0.00844 | 32.94766 |
| rs6769511 | 0.003182 | 462933 | 6.00E-20 | 0.000348 | 3 | 1.86E+08 | ukb-b-14609 | C | T | 0.315782 | 0.013437 | 83.60269 |
| rs17036160 | -0.00314 | 462933 | 4.70E-10 | 0.000504 | 3 | 12329783 | ukb-b-14609 | T | C | 0.117453 | -0.00915 | 38.79154 |
| rs1496653 | -0.00292 | 462933 | 3.50E-13 | 0.000401 | 3 | 23454790 | ukb-b-14609 | G | A | 0.203441 | -0.01069 | 52.88123 |
| rs10001190 | 0.002637 | 462933 | 3.90E-15 | 0.000336 | 4 | 6284633 | ukb-b-14609 | G | A | 0.632112 | 0.011547 | 61.7352 |
| rs7376543 | -0.00232 | 462933 | 2.40E-09 | 0.000389 | 4 | 49310408 | ukb-b-14609 | G | T | 0.68871 | -0.00878 | 35.65491 |
| rs459193 | 0.002364 | 462933 | 1.90E-10 | 0.000371 | 5 | 55806751 | ukb-b-14609 | G | A | 0.746628 | 0.009362 | 40.57669 |
| rs17250977 | 0.004587 | 462933 | 2.50E-08 | 0.000824 | 5 | 14753745 | ukb-b-14609 | G | A | 0.040221 | 0.008186 | 31.02535 |
| rs74567345 | 0.005631 | 462933 | 6.20E-14 | 0.00075 | 5 | 1.02E+08 | ukb-b-14609 | C | T | 0.051008 | 0.011029 | 56.32074 |
| rs9273268 | 0.002274 | 462933 | 3.80E-08 | 0.000414 | 6 | 32614334 | ukb-b-14609 | C | T | 0.301139 | 0.008083 | 30.24787 |
| rs7756992 | 0.003197 | 462933 | 2.20E-18 | 0.000366 | 6 | 20679709 | ukb-b-14609 | G | A | 0.266365 | 0.012853 | 76.49245 |
| rs987237 | 0.002427 | 462933 | 8.40E-09 | 0.000421 | 6 | 50803050 | ukb-b-14609 | G | A | 0.179582 | 0.008465 | 33.17301 |
| rs849142 | -0.0024 | 462933 | 1.30E-13 | 0.000323 | 7 | 28185891 | ukb-b-14609 | C | T | 0.505093 | -0.01089 | 54.91427 |
| rs13266634 | -0.00254 | 462933 | 3.80E-13 | 0.00035 | 8 | 1.18E+08 | ukb-b-14609 | T | C | 0.309592 | -0.01067 | 52.74462 |
| rs2796441 | -0.00183 | 462933 | 2.50E-08 | 0.000327 | 9 | 84308948 | ukb-b-14609 | A | G | 0.418465 | -0.00819 | 31.0899 |
| rs7018475 | 0.002723 | 462933 | 1.80E-13 | 0.00037 | 9 | 22137685 | ukb-b-14609 | G | T | 0.257503 | 0.010817 | 54.1688 |
| rs10965246 | -0.0043 | 462933 | 4.30E-24 | 0.000425 | 9 | 22132698 | ukb-b-14609 | C | T | 0.176686 | -0.01488 | 102.4984 |
| rs11257655 | 0.002735 | 462933 | 6.50E-12 | 0.000398 | 10 | 12307894 | ukb-b-14609 | T | C | 0.208209 | 0.010094 | 47.16828 |
| rs34744311 | -0.00285 | 462933 | 1.60E-17 | 0.000334 | 10 | 94467287 | ukb-b-14609 | T | C | 0.377322 | -0.01252 | 72.56942 |
| rs1613295 | 0.002401 | 462933 | 2.40E-13 | 0.000328 | 10 | 80954789 | ukb-b-14609 | G | T | 0.578289 | 0.010768 | 53.68248 |
| rs34872471 | 0.008564 | 462933 | 1.10E-127 | 0.000356 | 10 | 1.15E+08 | ukb-b-14609 | C | T | 0.291831 | 0.03531 | 577.9145 |
| rs76550717 | -0.00279 | 462933 | 3.50E-10 | 0.000444 | 11 | 72428172 | ukb-b-14609 | G | A | 0.159023 | -0.00922 | 39.36549 |
| rs2237895 | 0.002498 | 462933 | 2.40E-14 | 0.000328 | 11 | 2857194 | ukb-b-14609 | C | A | 0.415681 | 0.011207 | 58.14839 |
| rs4752792 | 0.002092 | 462933 | 1.10E-10 | 0.000324 | 11 | 47815702 | ukb-b-14609 | A | G | 0.544491 | 0.009477 | 41.58554 |
| rs7482891 | -0.00217 | 462933 | 7.80E-11 | 0.000334 | 11 | 2197112 | ukb-b-14609 | G | A | 0.622096 | -0.00956 | 42.30376 |
| rs947791 | 0.002289 | 462933 | 5.50E-09 | 0.000392 | 11 | 65302893 | ukb-b-14609 | A | G | 0.217618 | 0.008572 | 34.01579 |
| rs67232546 | 0.002274 | 462933 | 1.10E-08 | 0.000398 | 11 | 1.28E+08 | ukb-b-14609 | T | C | 0.212505 | 0.008394 | 32.62158 |
| rs9669278 | 0.001894 | 462933 | 5.80E-09 | 0.000325 | 12 | 66374587 | ukb-b-14609 | C | T | 0.51868 | 0.008558 | 33.90873 |
| rs1215468 | -0.00293 | 462933 | 2.70E-16 | 0.000358 | 13 | 80707429 | ukb-b-14609 | G | A | 0.291405 | -0.01203 | 66.99346 |
| rs7177055 | 0.002249 | 462933 | 3.70E-10 | 0.000359 | 15 | 77832762 | ukb-b-14609 | A | G | 0.717474 | 0.009212 | 39.28867 |
| rs4932264 | -0.00222 | 462933 | 1.20E-09 | 0.000365 | 15 | 90422986 | ukb-b-14609 | C | T | 0.729643 | -0.00893 | 36.94926 |
| rs72802357 | -0.00405 | 462933 | 2.50E-11 | 0.000606 | 16 | 75243142 | ukb-b-14609 | T | C | 0.078087 | -0.00981 | 44.54956 |
| rs1421085 | 0.003524 | 462933 | 1.10E-26 | 0.00033 | 16 | 53800954 | ukb-b-14609 | C | T | 0.403451 | 0.015708 | 114.2529 |
| rs11658063 | -0.00265 | 462933 | 2.00E-15 | 0.000333 | 17 | 36103872 | ukb-b-14609 | G | C | 0.602828 | -0.01167 | 63.0711 |
| rs9957264 | -0.00263 | 462933 | 1.60E-09 | 0.000435 | 18 | 56881633 | ukb-b-14609 | A | C | 0.166478 | -0.00887 | 36.42313 |
| rs2009222 | 0.001898 | 462933 | 1.40E-08 | 0.000335 | 19 | 13034543 | ukb-b-14609 | C | T | 0.632367 | 0.008332 | 32.13917 |
| rs10420309 | -0.00189 | 462933 | 7.40E-09 | 0.000328 | 19 | 46150182 | ukb-b-14609 | G | A | 0.437532 | -0.0085 | 33.4369 |
| rs1800961 | 0.005366 | 462933 | 9.00E-09 | 0.000933 | 20 | 43042364 | ukb-b-14609 | T | C | 0.030964 | 0.008449 | 33.04536 |
| rs73188924 | 0.002174 | 462933 | 2.40E-08 | 0.00039 | 22 | 50788567 | ukb-b-14609 | A | C | 0.224797 | 0.008197 | 31.1092 |
